# Transcriptome analysis of the mobile genome ICE*clc *in *Pseudomonas knackmussii *B13

**DOI:** 10.1186/1471-2180-10-153

**Published:** 2010-05-26

**Authors:** Muriel Gaillard, Nicolas Pradervand, Marco Minoia, Vladimir Sentchilo, David R Johnson, Jan Roelof van der Meer

**Affiliations:** 1 Department of Fundamental Microbiology, University of Lausanne, Bâtiment Biophore, Quartier UNI-Sorge, 1015 Lausanne, Switzerland; 2 Current Address: Institute of Biogeochemistry and Pollutant Dynamics, ETHZ, Zürich, and Department of Environmental Microbiology, Eawag, Dübendorf, Switzerland

## Abstract

**Background:**

Integrative and conjugative elements (ICE) form a diverse group of DNA elements that are integrated in the chromosome of the bacterial host, but can occasionally excise and horizontally transfer to a new host cell. ICE come in different families, typically with a conserved core for functions controlling the element's behavior and a variable region providing auxiliary functions to the host. The ICE*clc *element of *Pseudomonas knackmussii *strain B13 is representative for a large family of chromosomal islands detected by genome sequencing approaches. It provides the host with the capacity to degrade chloroaromatics and 2-aminophenol.

**Results:**

Here we study the transcriptional organization of the ICE*clc *core region. By northern hybridizations, reverse-transcriptase polymerase chain reaction (RT-PCR) and Rapid Amplification of cDNA Ends (5'-RACE) fifteen transcripts were mapped in the core region. The occurrence and location of those transcripts were further confirmed by hybridizing labeled cDNA to a semi-tiling micro-array probing both strands of the ICE*clc *core region. Dot blot and semi-tiling array hybridizations demonstrated most of the core transcripts to be upregulated during stationary phase on 3-chlorobenzoate, but not on succinate or glucose.

**Conclusions:**

The transcription analysis of the ICE*clc *core region provides detailed insights in the mode of regulatory organization and will help to further understand the complex mode of behavior of this class of mobile elements. We conclude that ICE*clc *core transcription is concerted at a global level, more reminiscent of a phage program than of plasmid conjugation.

## Background

The acquisition of horizontally transferred genes plays an important role in prokaryotic evolution [1]. The colonization of new ecological niches is often enabled by the acquisition of foreign genes, which can be transmitted by a large variety of mobile genetic elements (MGE) present in individual members of the microbial community. In terms of evolutionary success, it is thus interesting to understand how different mobile DNA elements control their mobility and may adapt to their bacterial host [2].

Various classes of MGE are known, the most well-studied of which are plasmids and bacteriophages [3,4]. Plasmids, apart from certain exceptions such as the F-episome in *Escherichia coli*, generally occur as extrachromosomal DNA in the bacterial cell. An important aspect of their life-style, therefore, is to ensure replication, stability and maintenance in the host cell [5], and a variety of control mechanisms have evolved hereto [6]. Conjugative plasmids encode and orchestrate specific machineries to produce the transfer system dedicated to their own distribution (e.g., type IV secretion system) [7]. By contrast, temperate bacteriophages insert into the host's chromosome, where they can remain silent and are co-replicated with the host's DNA for many generations, or are eventually genetically defunctionalized. Feedback regulatory systems silence phage behaviour in the temperate form, but can very rapidly induce the lytic phase (e.g., upon SOS response), upon which thousands of phage particles are produced to commence a new infection cycle [8,9].

More recently, a large new class of DNA elements has been recognized that contributes importantly to bacterial genome evolution via horizontal gene transfer. Most of these have been detected by comparative genome sequencing and have in general been named 'genomic islands' (GEI) to portray their foreign character within the host genome [10]. Often, according to the functions encoded by the GEI, they were classified as pathogenicity, symbiosis, metabolic, secretion or resistance islands [11,12]. Recently we and others proposed that GEI should be considered an overarching group of elements comprising both phage-like, Integrative and Conjugative Elements (ICE), as well as conjugative transposons [10]. Although GEI are assumed to have been acquired via horizontal gene transfer, for most of them self-transfer has not been tested under experimental conditions. In some cases only GEI excision from its chromosomal location has been observed, which is presumed to be the first step in horizontal transfer [13]. A self-transferable GEI (e.g., ICE, conjugative transposons and other types) can move its excised DNA to a new host, where it can reintegrate with the help of an integrase enzyme at one or more specific insertion sites. GEI transfer can be mediated by conjugation or transduction, either by the element itself or via mobilization by another MGE. For some GEI the conjugation machinery closely resembles that of known plasmid-types, such as that of the SXT element of *Vibrio cholerae *[14] or the ICE*MlSymR7A *element of *Mesorhizobium loti *[15]. For others it is very distantly related to known plasmid conjugative systems, like for ICE*Hin1056 *of *Haemophilus influenzae*, suggesting them to be evolutionary ancient elements [16]. The findings that many GEI resemble phages by their integrase, but plasmids by their conjugative system [10], suggests they are evolutionary hybrids, which may have global control mechanisms reminiscent of both phages and plasmids. To better understand the global control of such evolutionary hybrid elements and the consequences of the element's behavior for its bacterial host, it would be helpful to have detailed information on their transcriptional organization and regulation, which is presently still very fragmented. The SXT-element, for example, displays a key regulator (SetR) similar to the phage λ CI repressor that is autocleaved upon SOS response, after which SXT transfer becomes strongly induced [17,18]. Preliminary regulation studies were also performed on ICE*Hin1056 *[16] and the *Pseudomonas aeruginosa *elements pKLC102 and PAGI-2 [19], but without attaining a global level.

**Figure 1:**
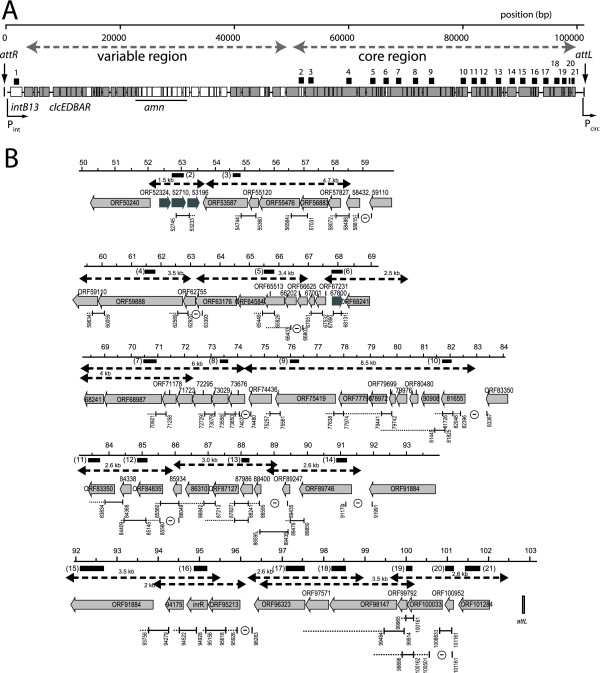
**Global gene organization of ICE*clc *and strategy for analysis of the core region transcriptional units**. A) Approximate locations of the ICE*clc *variable and core regions, with indication of gene functions known so far. Open reading frames are indicated by open (plus strand) or grey boxes (minus strand). Small numbered black stripes above point to the location of the probes used for macroblot hybridizations. B) Detailed gene structure of the core region with positions and results of RT-PCR analysis, and placement of transcript lengths (dashed lines) revealed by Northern analysis using the probes indicated as black numbered bars below the scale bar. RT-PCR indications are the following: stippled line indicates reverse transcribed regions. Solid line with two upright ends indicates the amplified region. A 'minus' within a circle indicates that no amplicon was obtained for that region. ORF numbering for ICE*clc *as in Genbank AJ617740.

Our group has been studying a mobile GEI in *Pseudomonas*, *Ralstonia *and *Burkholderia*, called the *clc *element or ICE*clc *[20]. ICE*clc *has a size of 103 kilobase-pairs (kbp) and is integrated into the chromosome at the 3' 18-bp extremity of one or more *tRNAy*^Gly ^genes by the help of an unusually long P4-type integrase [21-23]. The first half of ICE*clc *encodes two catabolic pathways involved in chlorocatechol (*clc *genes) and 2-aminophenol (*amn *genes) degradation [20] (Figure 1A). The second half contains a large set of syntenic genes that were defined as life-style 'core' for sixteen GEIs originating from different *Beta*- and *Gammaproteobacteria *[24]. Among other things, this core has been proposed to encode a type IV conjugative secretion system distantly related to that of ICE*Hin1056 *[16]. In addition, this part of ICE*clc *is assumed to encode the relaxosome complex needed for conjugation and was shown to bear a regulatory factor controlling excision and transfer [25,26]. ICE*clc *is transferred from *P. knackmussii *B13 as donor to e.g., *Pseudomonas putida *as recipient almost exclusively in stationary phase cultures with frequencies of self-transfer ≈ 10^-2 ^per donor. Self-transfer rates are highest in stationary phase cells grown with 3-chlorobenzoate and lower with fructose [27]. In line with this, expression of the promoter for the integrase is highest after growth on 3-chlorobenzoate, lower on fructose and essentially absent on glucose [26]. Because of the conservation of the ICE*clc *core region among different GEIs we were interested to study its transcriptional organization, as a further step towards the understanding of the life-style program of this class of mobile elements.

In order to resolve the global transcription network of ICE*clc *in *P. knackmussii *B13, we carried out a combined approach of Northern hybridizations, reverse-transcriptase polymerase chain reaction (RT-PCR), semi-tiling array hybridization and Rapid Amplification of cDNA Ends (5'-RACE). We detected fifteen transcripts, some of which were expressed to high levels in stationary phase cultures, but -- interestingly, not with all carbon sources.

## Results

### Transcriptional organization of the ICE*clc *core region

In order to analyze the transcriptional organization of the core region of ICE*clc*, we used a combination of conventional molecular techniques and semi-tiling micro-array analyses. The ICE*clc *core spans the region between nucleotide 50,000 until the left end of the element (position 102,843; ICE*clc *numbering, GenBank Accession Number AJ617740), and comprises the most conserved stretch among a number of closely related GEI [24,26]. Furthermore, it includes the integrase gene at the other side of ICE*clc *(Figure 1A).

**Figure 2:**
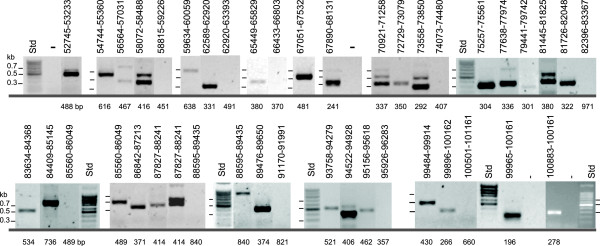
**Reverse transcriptase-PCR amplifications of the analyzed transcript connections indicated in Figure 1**. Numbers above amplicons indicate the examined region in ICE*clc *numbering; numbers below the calculated amplicon size. 'Minuses' are negative control reactions with PCR only without reverse-transcriptase step to verify DNA contamination. Different panels are reactions run on the same gel but not necessarily in consecutive lanes. Electronic images were auto-leveled and relevant lanes were placed side-by-side using Adobe Photoshop CS3. Std, DNA size standard (in kilobase-pairs, kb). At least one negative control was performed on every batch of purified RNA.

Figure 1 schematically presents the analysis of intergenic regions in the ICE*clc *core region, whilst combined RT-PCR results are shown in Figure 2. RT-PCR provided a first view of potentially linked polycistronic mRNAs. In a number of cases the clear absence of RT-PCR amplicons coincided with distinct large intergenic regions predicted from the nucleotide sequence (Figure 1B), whereas in other cases gaps were rather unexpected with respect to the close gene organization and could point to mRNA cleavage sites rather than new transcription start sites. This occurred, for example, in the regions between ORFs 62755-63176 (overlapping ORFs), ORFs 66202-66625 (12 bp intergenic region) and ORFs 73676-74436 (139 bp intergenic region, Figure 1, 2).

**Figure 3:**
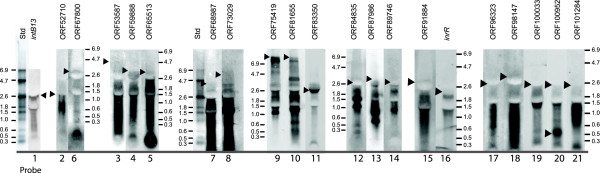
**Compiled Northern analysis of transcript sizes in the ICE*clc *core region on RNA isolated from cells grown to stationary phase on 3-chlorobenzoate**. Probe used in hybridization for a respective panel is indicated as the ORF number above and the probe number below, corresponding to the indications in Figure 1. Black triangles point to the largest size determined for the hybridizing transcript. Size indications for each panel in kilobases, based on an RNA size ladder. Electronic images were auto-leveled and relevant lanes were placed side-by-side using Adobe Photoshop CS3.

On top of the RT-PCR analysis we mapped the length of detectable transcripts by Northern hybridizations of RNA isolated from *P. knackmussii *B13 cultures grown to stationary phase on 3-chlorobenzoate (Figure 3). Arguably, Northern hybridizations do not always produce clear-cut signals and often show multiple bands indicative for mRNA degradation or processing, but for most of the transcript sizes and positions proposed by RT-PCR analysis supporting evidence was provided by Northerns (Figure 1, 3). Even the breakpoints detected between ORFs 62755-63176 coincided with two detectable transcripts of around 3.5 kb that could be positioned around the gap (Figure 1). The longest detected transcript seems to be formed by an estimated 8.5 kb polycistronic mRNA that would start upstream of ORF81655 and ending at ORF74436. It is possible, as we will argue below, that this transcript is actually synthesized as a much longer one, but cleaved somewhere in the area of the gap identified by RT-PCR between ORF73676 and 74436. The downstream part would be formed by a 6 kb mRNA that was detectable by probes for the ORFs 68987 and 73029 (Figure 3). Although a -10 promoter region was predicted upstream of ORF73676 by bioinformatic analysis, several others were predicted in this 8.5 kb region as well (see below and Table S1). Therefore, promoter prediction was not sufficiently accurate to support or refute the hypothesis for the 8.5 and 6 kb regions being transcribed as a single polycistronic mRNA.

### Micro-array analysis assisted transcript mapping

**Figure 4:**
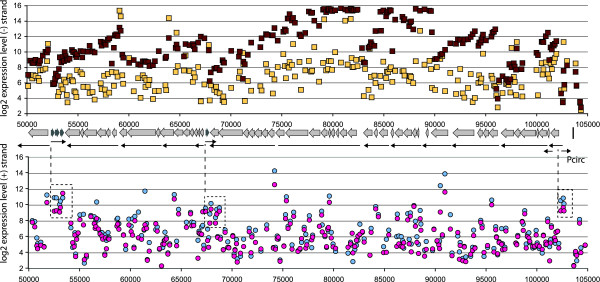
**Transcriptome of the ICE*clc *core region**. Shown is a compilation of micro-array hybridizations with minus- (top image) and plus-strand located probes (bottom image), both for exponential (yellow squares and blue circles) and stationary phase cultures (dark squares and pink circles). Data points are mean hybridization signals (on log2-scale) from six replicate probes per array, averaged over three replicate arrays.). X-axes, position numbering on ICE*clc*. Middle part, representation of the gene locations in the ICE*clc *core region (block arrows), and the size and position of the transcripts concluded from RT-PCR and Northerns (Figure 1-3).

To complement the RT-PCR and Northern analyses, we hybridized Cy3-labeled cDNA synthesized from total RNA isolated from *P. knackmussii *B13 cultures during exponential growth on 3-chlorobenzoate and during the following stationary phase, to custom-designed semi-tiling microarrays for ICE*clc*. The semi-tiling array contained a 50-mer probe at approximately every 200 bases over the whole length of ICE*clc *and for both strands, each in sixfold replicate on the array. We expected that a semi-tiling array format would permit us to map the position of ICE*clc *transcripts in a complementary way to the conventional molecular analysis, which would help to reinforce the conclusions drawn on the transcriptional organization of the ICE*clc *core. Figure 4 shows an overlay of the core gene organization and RT-PCR plus Northern derived transcriptional organization with the average micro-array hybridization signals per probe on the plus- and the minus-strand of the ICE*clc *core region, whilst Table 1 summarizes the transcript details across all three methods. Very strikingly, most of the predicted transcripts follow a clear 5'-3' decrease in signal intensity, the slopes of which were different for each transcript region (see, for example, the region for the long transcript proposed between position 82,000 and 68,000). We think the 5'-3' decrease in intensity may partially be caused by the fact that more transcripts are formed near the transcription start, which perhaps are incompletely finished, or by preferential 3'-end degradation. This effect has been noted by others using tiling approaches for transcript determination [28]. Different slopes may be the result of varying mRNA stability and processing speed.

**Table 1:** Summary of ICEclc core transcripts.

Transcript	**Strand**^**a**^	**Size on Northern**^**b**^	**RT-PCR**^**c**^	**Promoter**^**d**^	**Log2 Stat-Expo Ratio**^**e**^
*intB13*	+	2.5	+	102,729 (Pcirc)	3.1 ± 1.0
ORF50240	-	ND (1.8)	ND		1.6 ± 0.6
52324-53196	+	1.5 (1.2)	+	51,218	-1.2 ± 0.4
53587-58432	-	4.7 (5.3)	+	58,771	4.2 ± 1.4
59110-62755	-	3.5 (4.0)	+	63,191	2.6 ± 1.3
63176-66202	-	3.5 (3.4)	+	66,976	2.3 ± 1.7
66625-67231	-	ND (1.0)	+	67,610	5.8 ± 2.2
67800	+	2.6 (0.5)	+	67,817	-0.9 ± 0.2
68241-81655	-	4-6	+		4.0 ± 1.7
		8.5 (14.3)	(exc. 73676-74436)		5.7 ± 1.6
83350-84835	-	2.6 (2.3)	+		6.3 ± 1.6
85934-88400	-	3.0 (2.7)	+	89,109	6.5 ± 0.8
89247-89746	-	2.5 (2.1)	+		2.2 ± 1.9
91884-95213	-	3.5/2 (4.1)	+	96,204 (RACE)	5.6 ± 1.5
96323-100033	-	2.5-3.5 (4.5)			2.1 ± 1.6
100952	-	0.5	+		ND
100033-101284	-	2.6 (2.0)	+	102,270 (RACE)	2.0 ± 0.2

a) plus strand is same orientation as *intB13*.

b) in kilobase observed; within brackets, size calculated from sequence.

c) ORF connections detected by reverse-transcriptase PCR on RNA from strain B13 during stationary phase after growth on 3-chlorobenzoate.

d) Predicted location from bioinformatic analysis or observed by 5'RACE. Position according to numbering of AJ617740.

e) Log2-average ratio of hybridization intensities over all microarray probes covering the presumed transcript during stationary phase versus exponential phase on 3-chlorobenzoate.

Semi-tiling array hybridizations confirmed most of the proposed transcripts, including breakpoints, where the slope of the decrease in hybridization intensity as a function of probe position changed abruptly (e.g., regions around position 63,000 and 86,000). An exception here was the RT-PCR detected breakpoint in between ORFs 73676 and 74436, where micro-array hybridizations did not show any aberrant change in slope of signal decrease. From this, therefore, we conclude that the long transcripts of 8.5 and 6 kb mentioned above actually originate from one 14.5 kb-long polycistronic mRNA starting at ORF81655 and ending downstream of ORF68241. This transcript would then be rapidly processed in the indicated breakpoint area, although this should be confirmed by alternative techniques.

For one other region the pattern of 5'-3' decreasing slope did not match the hypothesis of a single transcript predicted from RT-PCR and Northern. This occurred in the area around 92,000 to 96,000 where RT-PCR had predicted a continuing transcript covering a four-gene cluster including ORF91884 (putatively encoding a DNA topoisomerase) [20], ORF94175 (putative single-strand DNA binding protein), *inrR *(the proposed IntB13 activator) [26] and ORF95213 (hypothetical protein). Indeed, Northerns had already suggested two transcripts here, not completely covering the whole region (Figure 1 and 3), and also tiling array hybridizations showed two or even three differently 'sloped' hybridization patterns. Therefore, it might be that there is read-through from ORF94175 into ORF91884, producing the detected RT-PCR connection, but an additional promoter upstream of ORF91884 does not seem unlikely (Table S1).

Whereas most of the genes in the ICE*clc *core region are organized on the minus strand (with respect to the *intB13 *gene, Figure 1), four genes are oriented on the plus strand. In general, hybridization signal intensities on micro-array for plus strand probes were lower than for the minus strand (Figure 4), but the signals coming from the small cluster of three genes at around 53,000 and the single ORF67800 can be discerned on micro-array as being significantly above local background.

### Carbon substrate dependent expression of ICE*clc *core genes

Micro-array hybridizations clearly demonstrated that most of the core genes on the minus strand are upregulated in stationary phase conditions (Table 1, Figure 4), with -- fold changes ranging from 2^2 ^(e.g., for ORF50240 or the cluster of genes between 96,000 and 100,000) to 2^7 ^(e.g., ORF81655). RNAs from a larger number of different growth conditions were hybridized in dot-blot format using digoxigenin-labeled probes representative for all proposed transcripts (Tables 2 and 3). This showed that the expression of the highly abundant core transcripts represented by ORF81655, ORF87986 and ORF84835 (Table 2) actually started in the first twelve hours after reaching stationary phase and then increased continuously further up to 72 h. In contrast, transcription from the three plus strand ORFs 52324-53196 seemed to 'peak' in very early stationary phase, but then successively decreased (Table 2). Hybridizing blotted RNAs from *P. knackmussii *B13 grown to stationary phase on different carbon substrates showed, interestingly, that the three transcripts 68241-81655 (represented by probes 7, 8, 9 and 10), 83350-84835 (probes 11 and 12), and 85934-88400 (probe 13) were highly induced only in stationary phase cells that had been cultured with 3-chlorobenzoate or fructose, but not at all with succinate or glucose (Table 3). Highest induction of the ICE*clc *core region genes in stationary phase cells grown with 3-chlorobenzoate is in agreement with previous experiments that showed the highest proportion of excised ICE*clc *and highest ICE*clc *transfer rates in cells cultured on 3-chlorobenzoate to stationary phase [26,27].

**Table 2:** ICEclc core gene transcript abundance in *P. knackmussii *B13 cultures grown with 3-chlorobenzoate as a function of growth phase as quantified by macroblot hybridization.

		expo		e-stat		12 h		24 h		36 h		48 h		72 h	
		
Probes	Probe number	mRNA^a^	Std Dev^b^	mRNA	Std Dev	mRNA	Std Dev	mRNA	Std Dev	mRNA	Std Dev	mRNA	Std Dev	mRNA	Std Dev
			(%)		(%)		(%)		(%)		(%)		(%)		(%)
*intB13*	*1*	**4.5**	11.2	**4.3**	12.9	**4.6**	15.6	**5.1**	28.5	**3.2**	5.3	**3.4**	0.9	**3.5**	14.6
ORF52710	2	**21.3**	46.5	**29.6**	8.7	**17.8**	3.4	**9.3**	39.9	**7.8**	53.8	**12.6**	18.6	**6.4**	41.8
ORF53587	3	**4.2**	30.2	**2.9**	25.9	**2.6**	27.1	**1.7**	37.3	**3.4**	11.9	**3.1**	20.4	**1.4**	12.2
ORF59888	4	**18.6**	33	**20**	7.5	**14.7**	18.9	**8.4**	32.3	**16.8**	23.9	**22.4**	9.3	**14.6**	43.4
ORF65513	5	**17.3**	19.4	**17.1**	0.8	**16.7**	10.3	**13.4**	9.9	**11.8**	9.5	**13.5**	2.4	**12.4**	10.7
ORF67800	6	**16.6**	2.7	**12.4**	26.1	**10.1**	11.6	**8**	12.9	**14.6**	4.3	**12.6**	10.7	**8.5**	16.6
															
ORF68987^c^	7	**2.1**	4.3	**1.7**	8.2	**1.2**	30.1	**0.8**	12.9	**1.7**	6.5	**1.5**	4.3	**1**	22.3
ORF73029	8	**2.5**	20.8	**1.4**	15	**2.1**	18.5	**2.6**	15	**2**	14.6	**2.2**	2.3	**1.5**	10.4
ORF75419	9	**7.5**	18.1	**4.5**	7.6	**8.7**	0.4	**11.1**	32	**14**	27.1	**20.5**	9.4	**28**	31.6
ORF81655	10	**10.2**	30.1	**6.4**	35.8	**104**	4.8	**168**	24.5	**113**	24.3	**191**	14.5	**177**	10.9
															
ORF83350	11	**3.3**	18.9	**1.7**	7	**0.9**	17.8	**0.9**	26.1	**0.9**	3.5	**0.9**	5	**0.9**	5.6
ORF84835	12	**0.4**	14.4	**0.3**	38.3	**7.4**	53.2	**9.5**	7.7	**20.6**	28.9	**28.1**	29.5	**29.8**	22.8
															
ORF87986	13	**5**	1	**4.6**	39.3	**41.1**	16.1	**64.5**	7.2	**41.3**	12.9	**54.9**	22.1	**100**	11.5
ORF89746	14	**12.9**	34.1	**12.9**	10.4	**4.9**	12	**2.2**	41.2	**1.3**	44.9	**3.4**	28.9	**3.2**	19.8
															
ORF91884	15	**3.3**	11.7	**2.1**	25.6	**2.4**	14.7	**3**	32.4	**3.1**	14	**3.2**	11.3	**4.1**	5.2
*inrR*	*16*	**8.3**	11.9	**6.4**	10.5	**7.4**	13.1	**4.5**	11.6	**5.1**	18.8	**7.2**	0.6	**5.9**	12
															
ORF96323	17	**3**	13	**1.7**	13.3	**1.1**	5.5	**1**	2.8	**1.3**	12.8	**1.1**	14.4	**1.2**	13.2
ORF98147	18	**1.1**	10.7	**0.5**	14.2	**0.4**	4.6	**0.5**	5.9	**0.5**	8.3	**0.4**	2.4	**0.4**	0.9
ORF100033	19	**30.6**	4	**23**	4.3	**26**	8.7	**12.3**	16.7	**19.6**	14.3	**20.4**	22.4	**21.5**	16.3
															
ORF100952	20	**1.4**	13.2	**3.7**	31.2	**2.8**	4.9	**1.8**	3.1	**1.7**	58.5	**3.5**	30.8	**6.7**	13.4
ORF101284	21	**3.7**	18.9	4.5	10.1	**2.1**	5.8	**1**	23.1	**1.9**	9.7	**1.5**	11	**1.3**	11.5

a) mRNA is the average calculated amount of mRNA copies × 10^8 ^per μg of total RNA from triplicate determinations. This value corresponds approximately to the calculated number of mRNA of this transcript per cell. Expo, exponential phase; e-stat, early stationary phase (6 h).

b) standard deviation from the average mRNA, expressed in percentage.

c) ORFs surrounded by a common border are on the same transcript.

**Table 3:** Quantification of ICEclc core gene expression by dot-blot hybridization in strain B13 grown on different carbon substrates.

	Exponential phase				After 24 h at stationary phase							
		
	3-chlorobenzoate		succinate		3-chlorobenzoate		succinate		fructose		glucose	
						
Probe number and probe	**mRNA**^**a**^	**Std Dev**^**b**^	mRNA	Std Dev	mRNA	Std Dev	mRNA	Std Dev	mRNA	Std Dev	mRNA	Std Dev
		(%)		(%)		(%)		(%)		(%)		(%)
1) *intB13*	**4.5**	11.2	**7.3**	13.1	**5.1**	28.5	**4.1**	11.2	**4.4**	51.7	**3.2**	8.1
2) ORF52710	**21.3**	46.5	**19.7**	16.9	**9.3**	39.9	**9.6**	8	**5.1**	42.7	**9.4**	30.6
3) ORF53587	**4.2**	30.2	**3.6**	0.1	**1.7**	37.3	**1.7**	21.1	**2**	0.4	**1.9**	2.6
4) ORF59888	**18.6**	33	**16.9**	2.3	**8.4**	32.3	**12.9**	18.6	**16.8**	7.3	**23.8**	15.9
5) ORF65513	**17.3**	19.4	**19.5**	2.8	**13.4**	9.9	**12.7**^**‡**^	5.3	**13.8**	7.6	**13.8**	11
6) ORF67800	**16.6**	2.7	**16.6**	5.5	**8**	12.9	**12.7**	18.3	**11.6**	33.7	**17.9**	38.6
7) ORF68987	**2.1**	4.3	**2.1**	11	**0.8**	12.9	**0.8**	0.2	**1.3**	13.8	**1.1**	11.7
8) ORF73029	**2.5**	20.8	**2.9**	12.6	2.6*	15	**0.9**^**‡**^	18.2	**1.4**	6.7	**1.1**	4.2
9) ORF75419	**7.5**	18.1	**7.3**	6.8	**11.1**	32	**3**^**‡**^	3.9	**3.9**	3.3	**2.8**	5.4
10) ORF81655	**10.2**	30.1	**18.7**	36.6	168*	24.5	**6.3**	2.7	45.7*	3.6	**9.2**	27
11) ORF83350	**3.3**	18.9	**2.8**	16.5	**0.9**	26.1	**0.5**	37.3	**0.5**	14.5	**0.4**	10.2
12) ORF84835	**0.4**	14.4	**0.3**	16.3	9.5*	7.7	**0.3**	25.8	**1.7**	16.1	**0.3**	1.5
13) ORF87986	**5**	1	**5.2**	0.1	64.5*	7.2	**5.5**^**‡**^	0.4	**14.2**	26.9	**5.5**	2
14) ORF89746	**12.9**	34.1	**24.4**	19.8	**2.2**	41.2	**2.1**	17.3	**2.1**	36.7	**0.5**	15.4
15) ORF91884	**3.3**	11.7	**4.5**	3	**3**	32.4	**1.6**^**‡**^	3.2	**2.3**	33.1	**1.1**	5.9
16) *inrR*	**8.3**	11.9	**8.2**	21.3	**4.5**	11.6	**4**	7.5	**6.4**	8.1	**4.9**	39.7
17) ORF96323	**3**	13	**5.3**	27.1	**1**	2.8	**1.8**	35.6	**0.9**	53.2	**1.1**	31.8
18) ORF98147	**1.1**	10.7	**1.5**	5.1	**0.5**	5.9	**0.4**^**‡**^	7	**0.4**	3.7	**0.4**	1.7
19) ORF100033	**30.6**	4	**40.4**	20.2	**12.3**	16.7	**17.6**	18	**22.9**	6.4	**22.2**	30.2
20) ORF100952	**1.4**	13.2	**2.2**	22.2	**1.8**^**§**^	3.1	**0.9**	1.7	**1.9**	7.9	**0.9**	29.4
21) ORF101284	**3.7**	18.9	**3.2**	6.9	**1**	23.1	**1**^**‡**^	7.9	**1.1**	1.9	**1.2**	14.5

a) mRNA is the average calculated amount of mRNA copies × 10^8 ^per μg of total RNA from triplicate determinations. This value corresponds approximately to the calculated number of mRNA of this transcript per cell.

b) standard deviation from the average mRNA, expressed in percentage.

c) *, values statistically significantly different to the cells cultivated with other carbon sources. ^§^, values statistically significantly different to the cells cultivated with succinate or glucose. ‡, values statistically significantly different between exponential phase and stationary phase. P ≤ 0.05 in pairwise Student's T test.

**Figure 5:**
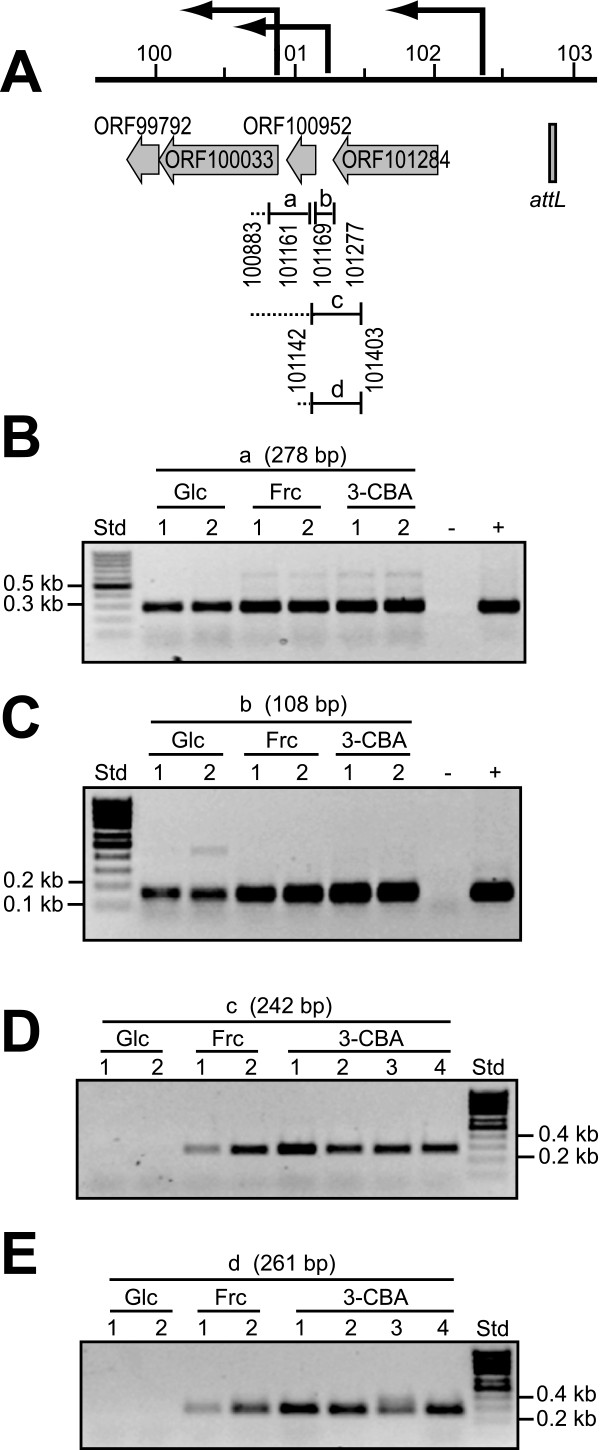
**Carbon substrate-dependent transcript linkage in the region at the outermost ICE*clc *left end**. A) Gene organization, reverse-transcribed regions and PCR amplicons. Arrows to the left point to inferred promoters. B) RT-PCR results for amplicon (a). C) idem for amplicon (b). D) Amplicon (c). E) Amplicon (d). All RNAs sampled from cultures during stationary phase after growth on the indicated carbon source. Glc, glucose. Frc, fructose. 3-CBA, 3-chlorobenzoate. Numbers below point to independent replicate reactions. -, PCR but without RT-step.+, PCR on B13 genomic DNA as template.

Another gene that produced relatively high signals in dot-blot hybridizations was ORF100033, which urged us to analyze its expression more conspicuously by RT-PCR. Contrary to RNA isolated in stationary phase from 3-chlorobenzoate or fructose-grown cultures, consistently no RT-PCR product was obtained for the intergenic region between ORF100952 and ORF101284 on RNA from cells that had been cultivated with glucose (Figure 5, panels d and e). RNA isolated from all three substrate conditions did produce a smaller RT-PCR fragment directly upstream of ORF100952 (Figure 5B panel b), suggesting that an additional promoter exists that produces a transcript covering ORF100952 only. In fact, Northern hybridizations with a probe for ORF100952 produced an additional band of 0.5 kb length (Figure 3). The promoter located in front of ORF101284 might thus be specifically repressed after growth on glucose (and perhaps succinate), or specifically activated after growth on 3-chlorobenzoate and fructose.

### Promoter analysis

Results from 5'-RACE were not as conclusive as expected. Although various amplicons were produced from cDNA ends, only few matched the start region for transcripts detected by RT-PCR, Northern and micro-array. In contrast, the start site for the transcript covering *inrR *could be mapped in the region upstream of ORF95213 to a thymine located 25 nt upstream of the ORF95213 start codon. Interestingly, the corresponding -10 box (TGTCGATCCT) and -35 (TTGACT) are close to the proposed consensus sequence of σ^s ^and not σ^70^, suggesting it is controlled by RpoS [26]. This could explain a higher abundance of this transcript during stationary compared to exponential phase as seen on micro-array (Figure 4). 5'-RACE also produced a clear transcription start upstream of ORF101284 (position 102,270), which is in agreement to the other data and suggests a promoter being present at positions 102,283 to 102,288 (-10, TATTAC) and 102,300 to 102,305 (-35 region, TTGCAG). On the contrary, no 5'RACE product but a very weak product was obtained by primer extension in the region upstream of ORF81655, which located at ~250 bp upstream of the start codon (results not shown), even though this transcript was among the most abundant ones of the ICE*clc *core region (Figure 4). In a few other cases, bioinformatic searches identified promoter signatures which locate in regions where transcripts were deemed to start (Table 1, S1), but their nature remains to be experimentally verified.

## Discussion

By using a combination of semi-tiling micro-array hybridization and conventional techniques for transcription analysis, we obtained a highly detailed picture on the transcriptional organization of the ICE*clc *core region. To our knowledge, this is one of the first examples of tiling micro-array in combination with RT-PCR and Northern hybridizations to study transcriptional organization of mobile DNA elements, the only other one currently being a study on the plasmid pCAR1 of *P. resinovorans *[29]. We conclude from our results that such a combined approach can give excellent complementary data and retrieve details that either one of the typical transcription approaches alone cannot obtain. Except for a few locations, the results from all approaches on ICE*clc*'s transcriptome were mostly in agreement with each other, or critically supported omissions in each of them individually. Fourteen transcripts were detected by RT-PCR and Northern; one more was inferred from micro-array hybridization (ORF50240). Some transcripts seem clearly part of one larger but rapidly cleaved polycistronic mRNA (e.g, ORF68241-81655), whereas in one case (ORF59110-67231) three transcripts were consistently detected but gene organization suggests close linkage.

**Figure 6:**
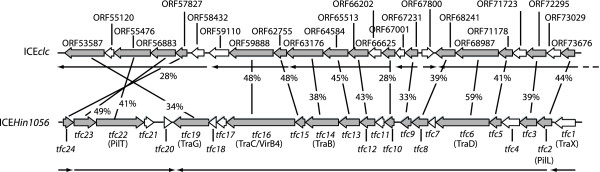
**Comparison of the *tfc*-like gene region on ICE*clc *with ICE*Hin1056 *from *H. influenzae***. Lines indicate percentage amino acid similarity between common genes (grey-shaded). Genes indicated in open arrows have no significant homologies among the two ICE. Arrows underneath point to the transcriptional organization in this region. Data on ICE*Hin1056 *redrawn from [16].

The importance of the ICE*clc *core gene region lays in its proposed control of the element's behavior: excision, self-transfer, maintenance and reintegration. Even though still only few ICE*clc *core genes have clear identifiable homology to known proteins (Additional file 1, Table S1), the region as a whole is largely conserved in a large collection of other GEI, underscoring its functional importance for life-style [23,24]. The 14 or 15 transcripts in the ICE*clc *core region, including a long 14.5 kb transcript (Figure 1, 4), is in the order of transcript numbers typically found for self-transfer and maintenance functions of conjugative plasmids (e.g., eight for R27 in *E. coli *[30], 14 for pCAR1 in *P. resinovorans *[29]). Four of the core transcripts (between ORF53587 and ORF73676) might code for a type IV secretion system (mating pair formation complex) similar to that of ICE*Hin1056 *from *H. influenza *(Figure 6, Additional file 1, Table S1) [16]. Furthermore, the ORF50240 product has weak homology to a relaxase and ORF91884 has a DNA topoisomerase domain, suggesting possible implication in a relaxosome complex involving the excised circular ICE*clc *form (Additional file 1, Table S1). A comparison with the ICE*Hin1056 *transcriptional organization in this area shows a number of differences, which are likely due to extensive gene arrangements during evolutionary divergence between the two elements (Figure 6). For example, the long ICE*Hin1056 *transcript covering the mating pair complex (PilL, TraB, TraD etc.), is interrupted on ICE*clc *by the reversely oriented ORF67800. The transcript containing ORF73676 (the presumed *pilL*) is not the start, but part of a much longer transcript starting at ORF81655 on ICE*clc*. Second difference between ICE*clc *and ICE*Hin1056 *relates to the large inversion of the genes *tfc21 *to *tfc24 *(Figure 6). ICE*Hin1056 *data suggested two transcripts in this region, with one being formed by the presumed regulatory gene *tfc24 *[16]. In contrast, on ICE*clc *ORF57827 (the homologue of *tfc24 *on ICE*clc*, Figure 6) is apparently the second gene of a six-gene transcript.

The relative abundance of transcripts in the region ORF50240 to ORF81655 of ICE*clc *was up to 64-fold (microarray) different between stationary and exponential phase (Figure 2 and 3, Table 1). If the postulate is correct that these genes would encode part of the type IV secretion system necessary for ICE*clc *transfer (i.e., the equivalent of the Mating Pair Formation or *mpf *complex in conjugative plasmids [6]), their induction would be much more pronounced than what is usual for plasmid conjugative systems. In most cases, the *mpf *genes are either weakly expressed or tightly regulated and inducible [6], the reason presumably being that expression of the conjugative apparatus is energy costly and could favor male-type specific phage infection. Tight control of the transfer genes of plasmids is often achieved by autoregulatory loops, such as the IncP-9 pWW0 plasmid *traA *and *mpfR *genes that control the relaxosome complex and *mpf *operons, respectively [31]. Also, the presumed genes involved in conjugative transfer of the IncP-7 plasmid pCAR1 in *Pseudomonas putida *and *P. resinovorans *are expressed at low and similar transcriptional level (without further specification) during growth on succinate or carbazole [29]. Induction of the putative conjugative system of ICE*clc *would thus be more similar to the type of induction found in the SXT element [18], which is a hybrid between phage-lambda type control and plasmid-like conjugation. However, none of the ICE*clc *functions has any significant sequence similarity to the SetR -- SetC -- SetD regulators of SXT, nor to the CI repressor from λ. The type of program that seems to be followed by ICE*clc *(integrase induction, excision, and global response of the core region) is indeed reminiscent of a phage program [9], but we conclude that control layers are different from phage type. The finding that basically eight of fifteen identified transcripts in the ICE*clc *core region are upregulated during stationary phase, suggests a coordinated global control mechanism, which is perhaps assisted by the stationary phase sigma factor RpoS. Indeed, some evidence for RpoS control was obtained from sequence motifs in the *inrR *promoter. It is interesting to speculate as to what would be the ecological or physiological advantage for ICE*clc *to become active during stationary phase. One hypothesis is that of the 'sinking ship': the element senses that its host survival (and therefore that of itself) is endangered and tries to escape to a more favorable host cell (even though this must be in its immediate vicinity). Even more intriguing is perhaps the carbon substrate-specific upregulation of ICE*clc *activity, which is highest after growth on 3-chlorobenzoate, less with fructose and very low with glucose or succinate as carbon sources. Upregulation of the ICE*clc *core region expression in stationary phase cells grown with 3-chlorobenzoate is in agreement with previous results showing increased activity of the integrase promoter [26], increased proportion of ICE*clc *excised DNA and increased ICE*clc *transfer rates [27]. Since it is assumed that during stationary phase cells have depleted their carbon source, the carbon source can no longer be directly be responsible for the activation, but somehow must have generated a 'memory' effect which triggers ICE*clc *response. In this light, the repression seen for transcription read-through from ORF101284 with glucose and succinate might point to a Crc-type regulation of catabolite repression in *Pseudomonas *[32,33], although for the time being no specific Crc binding motifs were detected in the ICE*clc *core region.

## Conclusions

In conclusion, we have identified fifteen transcripts covering the presumed core region for behavioral functions of ICE*clc*. Eight of those are concertedly upregulated during stationary phase, but only after previous growth of the cells on 3-chlorobenzoate or fructose, which explains previous results that have seen highest ICE*clc *transfer rates under such conditions [27]. The number and lengths of ICE*clc *transcripts is similar to that found for typical conjugative plasmid systems, yet the mode of global transcription control is more reminiscent for phage-type control. We thus conclude that the hybrid transcriptional control mode comprising both conjugative plasmid and phage strategies has been selected in mobile elements of the ICE*clc *group.

## Methods

### Growth conditions and harvesting

*P. knackmussii *B13, the original host of ICE*clc*, was cultivated in minimal medium (MM) based on the type 21C medium [34]. This MM was complemented with 3-chlorobenzoate, fructose, glucose (all at 10 mM) or 15 mM succinate, and the bacteria were grown at 30°C. Bacterial growth was assessed from culture turbidity at 600 nm (OD_600_). Cells were recovered during exponential phase (OD_600 _of 0.4) or early stationary phase (OD_600 _= 1.2), which was defined as the point where growth began to cease plus one period equivalent to the shortest generation time on that substrate. Bacteria were also recovered 12, 24, 36, 48 or 72 h after the beginning of the stationary phase. For RNA isolation, 100 ml of culture was immediately harvested by centrifugation (at 15,000 × *g *for 1 min at 4°C) and the supernatant was decanted. Cell pellets were resuspended in 4 ml RNAprotect Bacteria Reagent (QIAGEN GmbH). After 5 min incubation, the suspensions were centrifuged again (at 5,000 × *g *for 5 min at room temperature); the supernatant was discarded and pellets were stored at -80°C.

### RNA isolation

Prior to RNA extraction, pellets were slowly thawed, then resuspended in 0.5 ml TES buffer [10 mM Tris-HCl (pH 8.0), 1 mM EDTA, 100 mM NaCl], followed by addition of and mixing with 0.25 ml lysis solution [20 mM sodium acetate (pH 5.5), 1 mM EDTA, 0.5% SDS]. After that, the total RNA was further purified by the hot acid-phenol method as described previously [35]. RNA samples were purified from contaminating DNA by treatment with 50 U of DNase I (RNase free; Roche) during 1 h at 37°C. Finally, the RNA was dissolved in 50 μl diethylpyrocarbonate (DEPC)-treated water and quantified by absorbance at 260 and 280 nm on a NanoDrop spectrophotometer (Witec AG). The integrity of RNA was determined by agarose gel electrophoresis and the absence of DNA was verified by PCR.

### Reverse transcription PCR (RT-PCR)

Reverse transcription was made on RNA isolated from cultures grown with 3-chlorobenzoate, glucose or fructose, and harvested 24 h after the beginning of stationary phase. 0.5 μg of total RNA was denatured by heating at 65°C and reverse transcribed using the Omniscript RT kit (QIAGEN GmbH) following the instructions of the manufacturer, using primers listed in Additional file 1, Table S2. Primer designations refer to their exact position on ICE*clc *according to the numbering in AJ617740 (Genbank Accession number). 30 cycles of PCR amplification with the produced cDNA templates was performed with the HotStarTaq Master Mix kit (QIAGEN GmbH), using one tenth of volume from the reverse transcription reaction and 10 μM of a pair of specific primers (Additional file 1, Table S2). Amplification of regions between ORF94175 and *inrR *known to be co-transcribed served as positive control for the quality of the RT-PCR reaction. Finally, for each RNA sample, a PCR was performed without reverse transcriptase step, in order to control for the absence of DNA contamination.

### Mapping of transcriptional start sites

The 5' end of the transcript including *inrR *was mapped with the SMART RACE cDNA Amplification Kit (Clontech Laboratories, Inc.) according to the manufacturer's protocol. cDNA was synthesized from 0.5 μg RNA with the primer 95,129rv, which is located within the *inrR *region. After generation of RACE-Ready cDNA, a PCR and a nested PCR were performed by using the *inrR*-specific primer 95,156rv plus the Universal Primer A (UPM, Clontech), and the *inrR *primer 95,677rv plus the Nested Universal Primer A (NUP), respectively. Both PCR products were sequenced using a further *inrR *specific primer 95,790rv in the BigDye Terminator v3.1 cycle sequencing kit (Applied Biosystems), and were separated on ABI PRISM 3100 Genetic Analyzer (Applied Biosystems). A further successful mapping was deployed with 5'RACE on the transcript starting upstream of the most distal ICE*clc *ORF101284. 5'RACE reactions for the regions upstream of ORFs 58432, 66202, 73676, 81655, 88400, and 89746 did not produce specific fragments.

### Digoxigenin-labeled probe synthesis

DNA regions of between 126 and 560 bp of 21 selected ORFs from the *clc *element's core region (Figure 1) were amplified by PCR for probe synthesis (Additional file 1, Table S3). One of the PCR primers (reverse complementary to the targeted ORF) included the sequence for the promoter region of T7 RNA polymerase. Antisense digoxigenin-labeled RNA probes were then synthesized from ~1 μg of purified PCR product by using T7 RNA polymerase according to instructions of the suppliers (Roche Applied Science).

### Northern hybridization

20 μg of total RNA were incubated in 20 μl (total volume) of denaturation buffer (containing 1 M glyoxal, 25% *v/v *dimethylsulfoxide, 10 mM sodium phosphate, pH 7.0) for 1 h at 50°C. 100 ng of a digoxigenin-labeled RNA molecular weight marker I (0.3 -- 6.9 kb, Roche Diagnostics) was treated similarly. A volume of 0.2 μl of a 10 mg/ml ethidium bromide solution and 1 μl loading buffer (containing 50% sucrose, 15 mg/ml bromophenol blue in DEPC-treated H_2_O) were added to the samples at the end of the incubation period and mixed. Fragments were separated at 50 V on a 1% agarose gel in 10 mM sodium phosphate buffer (pH 7.0). RNA was subsequently transferred from gel onto Hybond N^+ ^nylon membrane (Amersham Biosciences) in 10 × concentrated SSC solution (containing 3 M NaCl and 0.3 M sodium citrate dissolved in demineralized H_2_O) with the help of the VacuGene XL system (Amersham Biosciences) for 3.5 h at a vacuum of 50 mbar. After transfer, RNA was fixed to the membrane with a UV crosslinker (CX-2000, UVP) at a dose of 0.3 J per cm^2^. Immediately before hybridization, the membrane was rinsed with 20 mM Tris-HCl (pH 8.0) at 65°C for 10 min to remove glyoxal. The hybridization was performed in DIG Hybridization buffer (Roche Diagnostics) for 15 h at 68°C. The washing steps and the immuno-chemiluminescent detection were done according to the supplier's instructions (Roche Diagnostics) using alkaline-phosphatase-conjugated anti-digoxigenin Fab fragments and CSPD as reagent for the chemiluminescence reaction. Light emission was detected on Hyperfilm ECL (Amersham Biosciences).

### Dot blot hybridization and relative mRNA quantification

Total RNA extracted from different growth phases and substrates were further analyzed for expression of genes from the core region of ICE*clc *in 96-well format dot-blot hybridization. RNA was isolated from three independent cultures of strain B13 grown with 3-chlorobenzoate at exponential phase, early-stationary phase, as well as at 12, 24, 36, 48 and 72 h after the beginning of stationary phase. Furthermore, duplicate cultures of B13 grown with glucose, fructose and succinate harvested after 24 h, and duplicate cultures grown on succinate in exponential phase were used for RNA purification as well. 15 μl Aliquots of dilutions containing 1, 0.3, and 0.1 μg denatured total RNA were dot-blotted using a 96-well manifold (Gibco Life Technologies) onto positively charged nylon transfer membranes (Hybond-N^+^, Amersham Biosciences AG). Different concentrations of denatured PCR products (2.5, 1, 0.5, 0.25, 0.1, 0.05, 0.025 and 0.01 ng) comprising the respective targeted ORF were included on the same blot. RNA was fixed to the membrane with a UV crosslinker before hybridization as described above. Films were scanned and spot intensities were calculated by densitometry using the Image Quant TL program (v2005, Molecular Dynamics, Sunnyville, USA) as grey intensity per standardized surface. The signal intensity of each spot was then compared to the standard curve of DNA dilutions on the same blot to calculate an 'equivalent number of DNA copies', and divided by the total amount of RNA in the spot to normalize to a value of 'equivalent number of copies per μg RNA'.

### Microarray design

A series of 950 non-overlapping 50-mer probes was designed to cover both coding and non-coding regions of the ICEclc sequence (Acc. No. AJ617740) at approximate distances of 200 bp. Probes were designed using the program Oligoarray version 2.1 [36] with a melting temperature range of 92 to 99°C and a probe GC content range of 52 to 72%. Probes were further designed to not cross-hybridize with gene products from the following potential host strains of the ICE*clc *element: *Burkholderia xenovorans *LB400 (Acc. No. CP000270-CP000272), *P. putida *F1 (Acc. No. CP000712), *P. putida *KT2440 (Acc. No. AE015451), *P. aeruginosa *PAO1 (Acc. No. AE004091), *Cupriavidus necator *JMP134 (Acc. No. CP000090-CP000093), and *Ralstonia metallidurans *CH34 (Acc. No. CP000352-CP000355). An additional 93 probes were designed to target housekeeping genes from the potential host strains and 8 probes were designed to target positive/negative controls (GFP, luciferase, and mCherry [37] transcripts). The microarray was manufactured by Agilent Technologies (Santa Clara, CA) in the 8 × 15,000 probe format and each unique probe was synthesized at six randomized spatial locations on the array. The microarray design has been deposited in the NCBI Gene Expression Omnibus http://www.ncbi.nlm.nih.gov/geo under accession number GSE20461.

### Microarray hybridization and analysis

Total RNA was isolated and purified from *P. knackmussii *B13 cultures during exponential growth on 3-chlorobenzoate and during subsequent stationary phase after 24 h as described above. For microarray hybridizations, cDNA was synthesized from total RNA and directly labeled with cyanine-3-dCTP using a modification of a protocol described elsewhere [38]. Briefly, each 50-μL reaction contained 10 μg of total RNA, 1.25 μg of random hexanucleotide primers (Promega), 100 μM each of unlabeled dATP, dGTP, and dTTP (Invitrogen), 25 μM of unlabeled dCTP (Invitrogen), 25 μM of cyanine-3-labeled dCTP (Perkin-Elmer), 25 U SUPERase•In (Ambion), and 400 U Superscript II reverse transcriptase (Invitrogen). Reactions were performed by heating at 42°C for 2 hours followed by 70°C for 10 min. RNA was then removed by adding 100 mM NaOH, heating to 65°C for 20 min, and neutralizing with 100 mM HCl and 300 mM sodium acetate (pH 5.2). Labeled cDNA products were purified using the MinElute PCR purification kit (Qiagen) and the quantity and incorporation frequency of cyanine-3-labeled dCTP were calculated using the microarray function on a NanoDrop Spectrophotometer. Sixty ng of labeled cDNA was then loaded onto each microarray, hybridized for 17 hours at 65°C, and washed and scanned as described for labeled cRNA in the One-Color Microarray-Based Gene Expression Analysis Manual (Agilent). The fragmentation step (heating to 60°C for 30 minutes) was omitted.

Hybridization signal intensities were quantified from microarray image scans using agilent feature extraction software version 9.5.3 (Agilent). Microarray data were normalized and globally scaled over the array using genespring gx software with the rma algorithm and quantile normalization [39,40]. Mean probe signals were calculated for each of the three biological replicates and were plotted against their position on the ICE*clc *sequence for both strands and for RNAs isolated during exponential and stationary phases. All microarray data have been deposited in the NCBI Gene Expression Omnibus http://www.ncbi.nlm.nih.gov/geo under accession number GSE20461.

### Bioinformatic tools

Putative promoters, terminators and transcription factor binding sites were predicted by using the BPROM and FindTerm programs on http://www.Softberry.com. The map of ICE*clc *was designed from SeqBuilder of the Lasergene software package (version 6.1.4, Dnastar, Inc).

## Authors' contributions

MG designed and performed transcription analysis. NP and MM performed microarray experiments. DJ designed probes for microarray and developed labeling and hybridization protocol. MG and VS carried out 5'RACE analysis. JvdM designed experiments and wrote the manuscript. All authors read and approved the final manuscript.

## Supplementary Material

Additional file 1 **Supplementary tables**. Location of ORFs in the ICE*clc *core region and bioinformatic predictions of protein function and transcription features. Primers used in this study. Probes produced for Northern hybridizations. Click here for file
