# Supplementary material for: Transcriptome analysis of the mobile genome ICEclc in Pseudomonas knackmussii B13
Source: BMC Microbiol. 2010 May 26;10:153. doi: 10.1186/1471-2180-10-153 (PMC2892462; doi:10.1186/1471-2180-10-153)
Supplement: Additional file 1 — Supplementary tables. Location of ORFs in the ICEclc core region and bioinformatic predictions of protein function and transcription features. Primers used in this study. Probes produced for Northern hybridizations. [file 1471-2180-10-153-S1.PDF]

Additional file 1 to:

Transcriptome analysis of the mobile genome ICE*clc* in *Pseudomonas knackmussii* B13

Muriel Gaillard, Nicolas Pradervand, Marco Minoia, Vladimir Sentchilo, David Johnson and Jan Roelof van der Meer*

Department of Fundamental Microbiology, University of Lausanne, Bâtiment Biophore, Quartier UNI-Sorge, 1015 Lausanne, Switzerland

*Corresponding author

Email addresses:

MG: muriel.gaillard@chuv.ch

NP: nicolas.pradervand@unil.ch

MM: marco.minoia@unil.ch

VS: vladimir.sentchilo@unil.ch

DJ: david.johnson@env.ethz.ch

JvdM: janroelof.vandermeer@unil.ch

- Additional file 1, **Table S1:** Location of ORFs in the ICE*clc* core region and bioinformatic predictions of transcription features.

- Additional file 1, **Table S2**: Primers used in this study

- Additional file 1, **Table S3 :** Probes produced for Northern hybridizations

Table S1: Location of ORFs in the ICE*clc* core region and bioinformatic predictions of transcription features.

ICEclc gene name	Gene name, product and % amino acid identity to ICEHin1056			Location on ICEclc	Transcript	Predicted -10 box	Predicted terminator	5'RACE
(attR)				60 - 77				
intB13				262 – 2235		148	1680	
ORF50240	(putative relaxase)			52087 – 50240	I	52361		
ORF52324				52324 - 52710	II	51218	52556	
ORF52710				52710 - 53168		52365		
ORF53196				53196 - 53573				
ORF53587	*tfc19*	TraG	34	53587 - 55104	III			
ORF55120				55120 - 55479				
ORF55476	*tfc22*	PilT	41	55476 - 56873				
ORF56883	*tfc23*		49	56883 - 57830				
ORF57827	*tfc24*		28	57827 - 58273				
ORF58432				58432 - 58926		58771	58471	
ORF59110				59110 - 59874	IV			
ORF59888	*tfc16*	TraC/V irB4	48	59888 - 62755		63191	62585	
ORF62755	*tfc15*		48	62755 - 63195		63855		
ORF63176	*tfc14*	TraB	38	63176 - 64594	V		64152	
ORF64584	*tfc13*		45	64584 - 65516				
ORF65513	*tfc12*		43	65513 – 66205				
ORF66202				66202 – 66612				
ORF66625	*tfc10*		28	66625 - 66984	VI	66976	66902	
ORF67001				67001 - 67234				
ORF67231	*tfc9*		33	67231 - 67614		67610	67501	

ORF67800				67800 - 68204	VII	67817		
ORF68241	*tfc8*		39	68241 - 68990	VIII			
ORF68987	*tfc6*	TraD	59	68987 - 71173		70182	70286	
ORF71178	*tfc5*		41	71178 - 71726		70851		
ORF71723				71723 - 72313		72183		
ORF72295	*tfc3*		39	72295 - 73014		72826		
ORF73029	*tfc2*	PilL	44	73029 - 73679				
ORF73676				73676 - 74296		74408	73693	
ORF74436				74436 - 75305			74710	
ORF75419				75419 - 77698		75373	76133	
ORF77798				77798 - 78907		78621	77243	
ORF78972				78972 - 79622				
ORF79699				79699 - 79959				
ORF79976				79976 - 80383		80141	80249	
ORF80480				80480 - 80812		80490		
ORF80908				80908 - 81597		80811	81030	
ORF81655				81655 - 82572		81326		~82822
ORF83350				83350 - 84192	IX	81845	83783	
ORF84338				84338 - 84691		84712	84674	
ORF84835				84835 - 85647			84825	
ORF85934				85934 - 86212	X		85850	
ORF86310				86310 - 87047		87066		
ORF87127				87127 - 87939				
ORF87986				87986 - 88378			88085	
ORF88400				88400 - 88612	XI	89109		
ORF89247				89247 - 89501		89491		
ORF89746				89746 - 91347		90548	91107	
ORF91884	(putative DNA topoisomerase)			91884 - 93896	XII	93515	92957	

ORF94175	(putative single stranded DNA binding protein)	94175 - 94615		93941	94002	
inrR		94689 - 95216		94614		
ORF95213		95213 - 95992		96024	95862	96017
ORF96323		96323 - 97567	XIII	97420	97156	
ORF97571		97571 - 98131		99656		
ORF98147		98147 - 99799		100105		
ORF99792		99792 - 100049		100552		
ORF100033		100033 - 100908		101378	100199	
ORF100952		100952 - 101164	XIV			
ORF101284		101284 - 102039	XV	102283		102270
attL		102826 - 102843		102757 (Pcirc)		

Table S1: Location of ORFs in the ICE*clc* core region and bioinformatic predictions of transcription features.

ICEclc gene name	Gene name, product and % amino acid identity to ICEHin1056			Location on ICEclc	Transcript	Predicted -10 box	Predicted terminator	5'RACE
(attR)				60 - 77				
intB13				262 – 2235		148	1680	
ORF50240	(putative relaxase)			52087 – 50240	I	52361		
ORF52324				52324 - 52710	II	51218	52556	
ORF52710				52710 - 53168		52365		
ORF53196				53196 - 53573				
ORF53587	*tfc19*	TraG	34	53587 - 55104	III			
ORF55120				55120 - 55479				
ORF55476	*tfc22*	PilT	41	55476 - 56873				
ORF56883	*tfc23*		49	56883 - 57830				
ORF57827	*tfc24*		28	57827 - 58273				
ORF58432				58432 - 58926		58771	58471	
ORF59110				59110 - 59874	IV			
ORF59888	*tfc16*	TraC/V irB4	48	59888 - 62755		63191	62585	
ORF62755	*tfc15*		48	62755 - 63195		63855		
ORF63176	*tfc14*	TraB	38	63176 - 64594	V		64152	
ORF64584	*tfc13*		45	64584 - 65516				
ORF65513	*tfc12*		43	65513 – 66205				
ORF66202				66202 – 66612				
ORF66625	*tfc10*		28	66625 - 66984	VI	66976	66902	
ORF67001				67001 - 67234				
ORF67231	*tfc9*		33	67231 - 67614		67610	67501	

ORF67800				67800 - 68204	VII	67817		
ORF68241	*tfc8*		39	68241 - 68990	VIII			
ORF68987	*tfc6*	TraD	59	68987 - 71173		70182	70286	
ORF71178	*tfc5*		41	71178 - 71726		70851		
ORF71723				71723 - 72313		72183		
ORF72295	*tfc3*		39	72295 - 73014		72826		
ORF73029	*tfc2*	PilL	44	73029 - 73679				
ORF73676				73676 - 74296		74408	73693	
ORF74436				74436 - 75305			74710	
ORF75419				75419 - 77698		75373	76133	
ORF77798				77798 - 78907		78621	77243	
ORF78972				78972 - 79622				
ORF79699				79699 - 79959				
ORF79976				79976 - 80383		80141	80249	
ORF80480				80480 - 80812		80490		
ORF80908				80908 - 81597		80811	81030	
ORF81655				81655 - 82572		81326		~82822
ORF83350				83350 - 84192	IX	81845	83783	
ORF84338				84338 - 84691		84712	84674	
ORF84835				84835 - 85647			84825	
ORF85934				85934 - 86212	X		85850	
ORF86310				86310 - 87047		87066		
ORF87127				87127 - 87939				
ORF87986				87986 - 88378			88085	
ORF88400				88400 - 88612		89109		
ORF89247				89247 - 89501	XI	89491		
ORF89746				89746 - 91347		90548	91107	
ORF91884	(putative DNA topoisomerase)			91884 - 93896	XII	93515	92957	

ORF94175	(putative single stranded DNA binding protein)	94175 - 94615		93941	94002	
inrR		94689 - 95216		94614		
ORF95213		95213 - 95992		96024	95862	96017
ORF96323		96323 - 97567	XIII	97420	97156	
ORF97571		97571 - 98131		99656		
ORF98147		98147 - 99799		100105		
ORF99792		99792 - 100049		100552		
ORF100033		100033 - 100908		101378	100199	
ORF100952		100952 - 101164	XIV			
ORF101284		101284 - 102039	XV	102283		102270
attL		102826 - 102843		102757 (Pcirc)		

Table S2: Primers used in this study

primer	sequence	location and position
Primers used for reverse transcription:		
53,360rv	5'-GACCAGCTTCACTACCCAT	reverse primer in ORF53196
54,608rv	5'-GCATCGATCTCCATACGCA	reverse primer in ORF53587
56,105rv	5'-AGAGGCTGCTACAACTGGT	reverse primer in ORF55476
57,883rv	5'-GCTCGCCATAGACCACGTA	reverse primer in ORF57827
58,625rv	5'-ACACGCTGTACGACGACAC	reverse primer in ORF58432
59,421rv	5'-GTGTGTGAGCATAGACCCA	reverse primer in ORF59110
62,351rv	5'-GTAGAGCTGAAGCACCCAG	reverse primer in ORF59888
65,306rv	5'-TCTGCACCCGTAGGCGTT	reverse primer in ORF64584
65,514rv	5'-CATGGGGTGCTTCCTCCTT	reverse primer in ORF65513
67,009rv	5'-GGAGGAAGAAACTCAGGAC	reverse primer in ORF67001
68,203rv	5'-CAGACCTTCAACCGTGCGT	reverse primer in ORF67800
70,762rv	5'-ACTCCAGCCGTTCCTCCA	reverse primer in ORF68987
72,557rv	5'-TTGACGAACACCGCCATAC	reverse primer in ORF72295
73,362rv	5'-GTCGTCGGCAGCGGCATA	reverse primer in ORF73029
75,187rv	5'-CACATGCGTTCCAGCCGA	reverse primer in ORF74436
77,206rv	5'-GCTTGATGAGCTTGACCAG	reverse primer in ORF75419
79,441rv	5'-TCCAGTCCCTGCTCGTCG	reverse primer in ORF78972
81,445rv	5'-CAGCTCCGCGTCCTTGAC	reverse primer in ORF80908
83,358rv	5'-CGCAGAAGCAGGCAATGG	reverse primer in ORF83350
84,350rv	5'-CACGACCTCCAACGACAAG	reverse primer in ORF84338
85,456rv	5'-GAGCTTGACGACGATGGCA	reverse primer in ORF84835
86,777rv	5'-CACCAACTACGCCTTCGC	reverse primer in ORF86310
87,711rv	5'-GATAGCTTTCATCCAATGCG	reverse primer in ORF87127
88,454rv	5'-GGCATAGCTGCGGAAGTA	reverse primer in ORF88400
89,417rv	5'-TGAAGCACATCGTTTCCTC	reverse primer in ORF89247
91,054rv	5'-GGCTACGCTGAACGACTG	reverse primer in ORF89746
95,129rv	5'-CTTTTAGAGAGGCCGCGTGTTCC	reverse primer in inrR
97,539rv	5'-GCTCGTGGTGCTGAGTCA	reverse primer in ORF96323
99,233rv	5'-GCTGATGTGCGACTGCTGTA	reverse primer in ORF98147
99,896rv	5'-CGGTCGAGCTGGTGTAGAT	reverse primer in ORF99792
100,843rv	5'-GAGGTTGGCTGCGGTGGT	reverse primer in ORF100033
101,054rv	5'-GGCACGCATCAGGTTGTAG	reverse primer in ORF100952
Primers used for PCR:		
52,745fw	5'-CTGTGTGATCGAGCAGTTG	forward primer in ORF52710
53,233rv	5'-AGGCGTTCTGCGGTGCTG	reverse primer in ORF53196
54,744rv	5'-TGCTGAAGGACTGCGACC	reverse primer in ORF53587
55,360fw	5'-CATCTACTTGGTCGGCAGT	forward primer in ORF55120
56,564rv	5'-AGCCGTTCGTGATGCCGT	reverse primer in ORF55476
57,031fw	5'-CCGTCCTGTCATCGTCCT	forward primer in ORF56883

58,072rv	5'-GCTCCGCTTCAATCCGATG	reverse primer in ORF57827
58,488fw	5'-CACATCGTCGTCGGTCTG	forward primer in ORF58432
58,815rv	5'-TGAAGATCCGTCTCTCCAG	reverse primer in ORF58432
59,266fw	5'-CCTGCTTGATCGCCAGAC	forward primer in ORF59110
59,634rv	5'-CATAGAGCGTCAGCGTGAA	reverse primer in ORF59110
60,059fw	5'-TGTGCCGCCAAGCCTCTA	forward primer in ORF59888
62,589rv	5'-GCCTGCTCGTCTGCCAAG	reverse primer in ORF59888
62,902rv	5'-CTGGAACTGGCGATAGACC	reverse primer in ORF62755
62,920fw	5'-GGTCTATCGCCAGTTCCAG	forward primer in ORF62755
63,393fw	5'-CCAGCAACGAAGCCGTAG	forward primer in ORF63176
65,449rv	5'-GCGGAGGACATGACCACT	reverse primer in ORF64584
65,829fw	5'-GCG GCA TCT ACG AAA TCC C	forward primer in ORF65513
66,433rv	5'-GATCGTGCGGAACACCCA	reverse primer in ORF66202
66,803fw	5'-TGGCATTGCTGGTTGTGGCGTC	forward primer in ORF66625
67,051rv	5'-TGCAGACGCCGAGGAACT	reverse primer in ORF67001
67,532fw	5'-CGATGGCGTTGGGCGAGT	forward primer in ORF67231
67,890fw	5'-GACAGTCTCGTTGAGTGGT	forward primer in ORF67800
68,131rv	5'-CTGATCAGTTCGGTCATCTC	reverse primer in ORF67800
70,921rv	5'-CGTCCTTGCTGGTCATCAC	reverse primer in ORF68987
71,258fw	5'-GTGTCCGGTGACGACTTG	forward primer in ORF71178
72,729rv	5'-AGCGCCGCCGTTCTTCAT	reverse primer in ORF72295
73,079fw	5'-ACCGCCGTGTTCCAGTCC	forward primer in ORF73029
73,558rv	5'-TGGTCGTTCATGGTCTGGT	reverse primer in ORF73029
73,850fw	5'-GCTGCTCACACTGGCTGG	forward primer in ORF73676
74,073rv	5'-AGCGTGTAGCGACCGTAG	reverse primer in ORF73676
74,480fw	5'-CAACTGGCACAGGTCGATC	forward primer in ORF74436
75,257rv	5'-TCAGGCGAGCATTGACGGT	reverse primer in ORF74436
75,561fw	5'-TGACCTGCCTTGAACTGATG	forward primer in ORF75419
77,638rv	5'-CGAGCAGTTCGCCCTGTA	reverse primer in ORF75419
77,974fw	5'-CTCGTCGTCAAAGGTGACA	forward primer in ORF77798
79,441rv	5'-TCCAGTCCCTGCTCGTCG	reverse primer in ORF78972
79,742fw	5'-CACTTCACTTCAACCCGCA	forward primer in ORF79699
81,445rv	5'-CAGCTCCGCGTCCTTGAC	reverse primer in ORF80908
81,726rv	5'-CAGATTGGTCGGCAGCGAT	reverse primer in ORF81655
81,825fw	5'-GTCTATCCGACGAGCCAG	forward primer in ORF81655
82,048fw	5'-TCGGTACTTCGACACCATC	forward primer in ORF81655
82,396rv	5'-GTCGAAGTAGCGGTAACTG	reverse primer in ORF81655
83,367fw	5'-TGCTTCTGCGGCGACTGA	forward primer in ORF83350
83,834rv	5'-CGAATCTTCAGGCTGGTC	reverse primer in ORF83350
84,368fw	5'-CTTGTCGTTGGAGGTCGTG	forward primer in ORF84338
84,409rv	5'-GCAAGATGCGGGTGAATAC	reverse primer in ORF84338
85,145fw	5'-CAGGTGCACGGGATTTCAC	forward primer in ORF84835
85,560rv	5'-ACACGCGGTACTTGAACGA	reverse primer in ORF84835
86,049fw	5'-GCGACATTGACGACTACGAA	forward primer in ORF85934
86,842rv	5'-ACAGGTTCAGTGCCATTCC	reverse primer in ORF86310
87,213fw	5'-GGCATCAACGACGAGCATT	forward primer in ORF87127
87,827rv	5'-GAGAAGTGAGCGGAGCCA	reverse primer in ORF87127

88,241fw	5'-CACCCGTGAAGGTCTGAAC	forward primer in ORF87986
88,595rv	5'-AGAGGCTTGCTGGGACAT	reverse primer in ORF88400
89,435fw	5'-GAGGAAACGATGTGCTTCA	forward primer in ORF89247
89,476rv	5'-CGGTCTTGCTTGGTGATGT	reverse primer in ORF89247
89,850fw	5'-CGCTACAAGGCAATCGGC	forward primer in ORF89746
91,170rv	5'-GGTAACGATGGGCGAGCA	reverse primer in ORF89746
91,991fw	5'-TGGTCGTGCAGTCGTTAC	forward primer in ORF91884
95,156rv	5'-GCATGAAGGCTTCCTGTTGCAGG	reverse primer in inrR
95,677rv	5'-CCAGGCTCAATGCTGCGGGTA	reverse primer in ORF95213
95,790rv	5'-TCGCTGTACGGGTCGTCTTGCTC	reverse primer in ORF95213
99,484rv	5'-TCTCCGACCAGAGTTCACGC	reverse primer in ORF98147
99,896rv	5'-CGGTCGAGCTGGTGTAGAT	reverse primer in ORF99792
99,914fw	5'-ATCTACACCAGCTCGACCG	forward primer in ORF99792
99,965rv	5'-TGACGTGCCAGCCTTCGC	reverse primer in ORF99792
100,162fw	5'-AGCCGCTCTCGACACCATG	forward primer in ORF100033
100,501rv	5'-GGCCATCTCCAGCAAGAC	reverse primer in ORF100033
100,883rv	5'-GTCGAGATGATGGATACAAC	reverse primer in ORF100033
101,142rv	5'-GGTAGTACAGGTATCTGCGA	reverse primer in ORF100952
101,161fw	5'-TCGCAGATACCTGTACTACC	forward primer in ORF100952
101,169rv	5'-TTTTTGGATCCGTCACTCCTGAACGCTCA	reverse primer in intergenic region between ORF100952 and ORF101284
101,277fw	5'-TTTTTCTGCAGCAAGCCCGAATCTGCGGT	forward primer in intergenic region between ORF100952 and ORF101284
101,403fw	5'-CAGGAGGCTATCGCTACC	forward primer in ORF101284

Table S3. Description of RNA probes for Dot Blot hybridization

Probe Name	Localisation	Size	Primer Name and Sequence
integrase	1,887 - 2,173	286	2,173rv-T7P: 5'-CTAATACGACTCACTATAGGGAGA / 1,887fw:
ORF52710	52,745 - 53,082	337	53.082rv-T7P: 5'-CTAATACGACTCACTATAGGGAGA / 52,745fw:
ORF53587	54,608 - 54,840	232	54,608rv-T7P: 5'-CTAATACGACTCACTATAGGGAGA / 54,840fw
ORF59888	61,503 - 61,820	317	61,503rv-T7P: 5'-CTAATACGACTCACTATAGGGAGA / 61,820fw
ORF65513	65,514 - 65,829	315	65,514rv-T7P: 5'-CTAATACGACTCACTATAGGGAGA / 65,829fw
ORF67800	67,842 - 68,203	361	68,203rv-T7P: 5'-CTAATACGACTCACTATAGGGAGA / 67,842fw
ORF68987	70,487 - 70,944	457	70,487rv-T7P: 5'-CTAATACGACTCACTATAGGGAGA / 70,944fw
ORF73029	73,362 - 73,640	278	73,362rv-T7P: 5'-CTAATACGACTCACTATAGGGAGA / 73,640fw
ORF75419	75,961 - 76,272	311	75,961rv-T7P: 5'-CTAATACGACTCACTATAGGGAGA / 76,272fw
ORF81655	81,726 - 82,048	322	81,726rv-T7P: 5'-CTAATACGACTCACTATAGGGAGA / 82,048fw
ORF83350	83,358 - 83,717	359	83,358rv-T7P: 5'-CTAATACGACTCACTATAGGGAGA / 83,717fw
ORF84835	84,867 - 85,145	278	84,867rv-T7P: 5'-CTAATACGACTCACTATAGGGAGA / 85,145fw
ORF87986	88,033 - 88,241	208	88,033rv-T7P: 5'-CTAATACGACTCACTATAGGGAGA / 88,241fw
ORF89746	90,898 - 91,187	289	90,898rv-T7P: 5'-CTAATACGACTCACTATAGGGAGA / 91,187fw
ORF91884	92,115 - 92,675	560	92,115rv-T7P: 5'-CTAATACGACTCACTATAGGGAGA / 92,675fw
inrR	94,886 - 95,204	318	94,886rv-T7P: 5'-CTAATACGACTCACTATAGGGAGA / 95,204fw
ORF96323	97,097 - 97,556	459	97,097rv-T7P: 5'-CTAATACGACTCACTATAGGGAGA / 97,556fw
ORF98147	98,203 - 98,528	325	98,203rv-T7P: 5'-CTAATACGACTCACTATAGGGAGA / 98,528fw
ORF100033	100,036 - 100,162	126	100,036rv-T7P: 5'-CTAATACGACTCACTATAGGGAGA / 100,162fw
ORF100952	100,952 - 101'161	209	100,952rv-T7P: 5'-CTAATACGACTCACTATAGGGAGA / 101,161fw
ORF101284	101'444 - 101'788	344	101,444rv-T7P: 5'-CTAATACGACTCACTATAGGGAGA / 101,788fw:
